# The Role of Focal Therapy in the Management of Localised Prostate Cancer: A Systematic Review

**DOI:** 10.1016/j.eururo.2013.05.048

**Published:** 2014-10

**Authors:** Massimo Valerio, Hashim U. Ahmed, Mark Emberton, Nathan Lawrentschuk, Massimo Lazzeri, Rodolfo Montironi, Paul L. Nguyen, John Trachtenberg, Thomas J. Polascik

**Affiliations:** a Division of Surgery and Interventional Science, University College London, London, UK; b Department of Urology, University College Hospitals NHS Foundation Trust, London, UK; c Department of Urology, Centre Hospitalier Universitaire Vaudois, Lausanne, Switzerland; d Department of Surgery, University of Melbourne; and Ludwig Institute for Cancer Research, Austin Hospital, Melbourne, Australia; e Department of Urology, Ospedale San Raffaele Turro, San Raffaele Scientific Institute, Milan, Italy; f Section of Pathological Anatomy, Polytechnic University of the Marche Region, School of Medicine, United Hospitals, Ancona, Italy; g Department of Radiation Oncology, Dana-Farber/Brigham and Women's Cancer Centre, Harvard Medical School, Boston, MA, USA; h Division of Urology, Department of Surgical Oncology, University Health Network; and Department of Surgery, University of Toronto, Toronto, ON, Canada; i Division of Urology, Department of Surgery, and Duke Cancer Institute, Duke University Medical Centre, Durham, NC, USA

**Keywords:** Brachytherapy, Cryotherapy, High-intensity focused ultrasound, Laser therapy, Photodynamic therapy, Prostate cancer

## Abstract

**Context:**

The incidence of localised prostate cancer is increasing worldwide. In light of recent evidence, current, radical, whole-gland treatments for organ-confined disease have being questioned with respect to their side effects, cancer control, and cost. Focal therapy may be an effective alternative strategy.

**Objective:**

To systematically review the existing literature on baseline characteristics of the target population; preoperative evaluation to localise disease; and perioperative, functional, and disease control outcomes following focal therapy.

**Evidence acquisition:**

Medline (through PubMed), Embase, Web of Science, and Cochrane Review databases were searched from inception to 31 October 2012. In addition, registered but not yet published trials were retrieved. Studies evaluating tissue-preserving therapies in men with biopsy-proven prostate cancer in the primary or salvage setting were included.

**Evidence synthesis:**

A total of 2350 cases were treated to date across 30 studies. Most studies were retrospective with variable standards of reporting, although there was an increasing number of prospective registered trials. Focal therapy was mainly delivered to men with low and intermediate disease, although some high-risk cases were treated that had known, unilateral, significant cancer. In most of the cases, biopsy findings were correlated to specific preoperative imaging, such as multiparametric magnetic resonance imaging or Doppler ultrasound to determine eligibility. Follow-up varied between 0 and 11.1 yr. In treatment-naïve prostates, pad-free continence ranged from 95% to 100%, erectile function ranged from 54% to 100%, and absence of clinically significant cancer ranged from 83% to 100%. In focal salvage cases for radiotherapy failure, the same outcomes were achieved in 87.2–100%, 29–40%, and 92% of cases, respectively. Biochemical disease-free survival was reported using a number of definitions that were not validated in the focal-therapy setting.

**Conclusions:**

Our systematic review highlights that, when focal therapy is delivered with intention to treat, the perioperative, functional, and disease control outcomes are encouraging within a short- to medium-term follow-up. Focal therapy is a strategy by which the overtreatment burden of the current prostate cancer pathway could be reduced, but robust comparative effectiveness studies are now required.

## Take Home Message

The functional outcomes of focal therapy are encouraging, but there is a lack of high-quality evidence of long-term disease-control outcomes. Although this strategy could decrease the treatment-related side effects of men requiring active treatment, further robust comparative effectiveness research is required.

## 1 Introduction

The advent of prostate-specific antigen (PSA) screening has led to stage, grade, and risk migration towards diagnosis of less aggressive prostate cancer (PCa). As a result, men with localised PCa and physicians who advise them face a difficult therapeutic dilemma: surveillance versus radical whole-gland therapy. The available evidence from randomised controlled trials (RCTs) demonstrates that there is little to no difference between these choices in terms of overall and cancer-specific survival after a median of 10 yr of follow-up [1]. In light of these findings, the patient's dilemma is made that much more profound by the significant rates of genitourinary and rectal side effects, which can occur despite technological improvements in surgery and radiation [2], [3], [4], [5].

Consequently, there has been interest in focal therapy. This tissue-preserving strategy has at its core the reduction of treatment-related toxicity by minimising damage caused to the prostate and adjacent structures while attempting to retain the benefits of treating cancer [6], [7], [8], [9]. This is an approach adapted by many other solid-organ malignancies, including renal, thyroid, breast, liver, and pancreas, but in which PCa has limited evidence and acceptance. Indeed, since whole-mount analysis of radical prostatectomy specimens has shown the presence of multiple foci of disease in most cases, the perception has been that whole-gland therapies are mandatory. However, new evidence suggests that the natural history of the disease is predominantly driven by the largest lesion with the highest grade, the so-called index lesion [10]. Therefore, targeted treatment delivered to the index lesion while sparing the rest of the gland may be a rational approach in men with intermediate- and low-volume, high-risk PCa that has disease suitable for a focal tissue-preserving approach. This proposition could make focal therapy achievable in the majority of men with localised PCa.

At the moment, any approach able to preserve part of the prostatic tissue (eg, “hockey stick” ablation, hemiablation, and focal ablation) is considered *focal therapy*. Many groups have published limited data on outcomes following focal therapy, and many others are actively engaged or considering prospective comparative effectiveness research in this area. It is an opportune time for a systematic review to evaluate the current evidence base and identify strengths and weaknesses and points of uncertainty about focal therapy to guide future trials.

## 2 Evidence acquisition

This systematic review was performed according to the Preferred Reporting Items for Systematic Reviews and Meta-Analyses (PRISMA) guidelines. We limited our systematic search to studies reporting on actual focal-therapy outcomes. We report on the following specific categories of data from the identified literature: (1) definition of the ideal candidate for focal therapy, (2) disease localisation, (3) identification of which lesions to target, (4) definitions of success and failure in focal therapy, and (5) morbidity and cancer-control outcomes after focal therapy.

Studies were identified by electronic search of Medline (through PubMed), Embase, Web of Science, and Cochrane Review databases from inception of the each respective database through 31 October 2012, with prespecified English language and human-studies restrictions. In addition, registered trials were retrieved from trials registries (ClinicalTrials.gov and the International Standard Randomised Controlled Trial Number). We conducted a search of ongoing trials to allow us to determine the current thinking on patient eligibility, disease localisation, and types of outcome measures that investigators in this area are currently using. The search strategy was as follows: “PCa” OR “prostatic neoplasms” AND “focal treatment” OR “focal therapy” OR “tissue-preserving/-preservation” OR “subtotal” OR “cryosurgery” OR “cryotherapy” OR “cryoablation” OR “high-intensity focused ultrasound ablation” OR “HIFU” OR “photodynamic therapy” OR “PDT” OR “laser therapy” OR “brachytherapy.”

RCTs, prospective development studies, and retrospective case series investigating ablative techniques to treat biopsy-proven PCa in a subtotal manner in the primary or salvage setting were included. Eligibility was reviewed separately by two reporters (M.V. and H.U.A.). In case of disagreement despite further discussion between the two authors, the senior author (T.J.P.) arbitrated. All selected articles were fully reviewed, and data extraction was predefined pro forma. Authors of included studies were contacted when one of the outcomes was not clearly or explicitly reported or when there were concerns about duplicate data sets; one reminder was sent for nonreplies. In cases where no reply was received, we chose not to report uncertain outcomes. When two or more series completely overlapped in time, only the largest series was reported; when the overlapping was partial over a limited time, all studies were reported, and the possible duplication of data was highlighted in the tables.

The primary end point was treatment-related side effects. We defined these in the following manner and differentiated them based on those reported by physicians and those using validated patient-reported questionnaires: leak-free continence, leak-free and pad-free continence, erections sufficient for penetration, and rectal toxicity (diarrhoea, bleeding, pain, rectourethral fistula). Functional outcomes were extracted from each study only when preoperative and postoperative data were available. In other words, only patients with normal function before treatment were considered. For instance, when calculating erectile function outcome, the denominator was represented by the men potent before the operation. Secondary end points were failure defined by residual PCa in the treatment area proven by biopsy, overall complications, quality of life (QOL) outcomes, need for secondary local or systemic treatment, and mortality. Biochemical outcomes also were reported.

The following data were extracted from each study:

• Predefined eligibility criteria
• Participants, including sample size, age, D’Amico or National Comprehensive Cancer Network cancer Risk classification, PSA level, and Gleason grade
• Preoperative diagnostic tools, such as imaging and biopsy techniques used to localise disease
• Type of intervention, including ablation modality, type of focal therapy, type of anaesthesia, and length of hospital stay
• Follow-up duration
• Toxicity
• Cancer-control measures, including histology (divided into *for cause* and *protocol* biopsy based on whether biopsies were conducted on suspicion of failure or whether the protocol required biopsies in all men, respectively) and biochemical disease-free survival (using current, nonvalidated definitions)
• PSA kinetics
• Need for additional treatments
• Metastatic disease
• Mortality.

Trifecta outcomes (pad-free continence, erections sufficient for penetration with or without oral phosphodiesterase type 5 inhibitors [PDE5-Is], and disease control at last follow-up) were extracted where possible.

The design of each study was reported according to the Idea Development Evaluation Assessment and Long-term (IDEAL) recommendations for evaluation of surgical innovations, proposed by the Balliol Collaboration and based on the UK Medical Research Council guidelines for evaluating complex interventions [11]. The quality of studies was assessed according to the level of evidence for therapy [12].

## 3 Evidence synthesis

### 3.1 Assessment of study quality

**Fig. 1:**
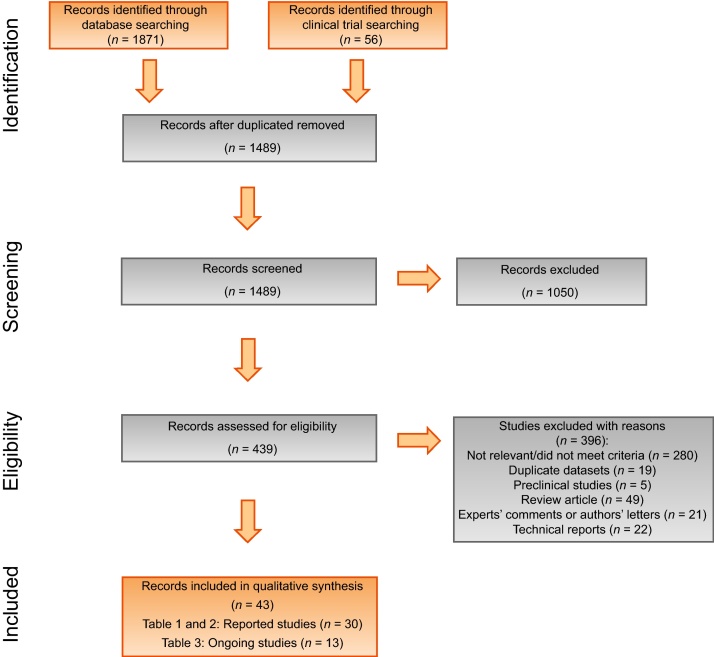
Preferred reporting items for systematic review and meta-analysis flowchart.

Overall, 43 studies were included; the selection process is displayed in Figure 1. The quality of the evidence is low to medium, with no study yielding a level of evidence >2b (Table 1, Table 2). Indeed, this classification system attributes the quality mainly according to the study design; therefore, only RCTs or systematic reviews of RCTs, which have not been performed in focal therapy, are classified at higher levels of evidence. Although this suggests that the results of this review should be interpreted with caution, it should be highlighted that several surgical techniques established in clinical practice were based on similar levels of evidence [13], [14].

**Table 1:** Target population with stage of evaluation and level of evidence of 25 studies using focal therapy in the primary setting

									Eligibility criteria				
Reference	Setting	IDEAL stage of evaluation	Previous treatment	Patients, no.	Age, yr	Preoperative biopsy	Preoperative imaging	Criteria for bDFS	Spatial location	PSA, ng/ml	Gleason score	Risk classification (D’Amico or NCCN)	Level of evidence
Madersbacher et al. [15]	Primary	1	NR	29	Mean: 64 (SD: 7.2)	NR	NR	NR	Unilateral	NR	NR	NR	2c
Zlotta et al. [16]	Primary	1	NR	15	NR	NR	NR	NR	Organ confined	NR	NR	NR	2c
Beerlage et al. [17]	Primary	1	NR	14	Mean: 62 (range: 55–69)	TRUS biopsy	MRI Bone scan	NR	NR	NR	NR	NR	2c
Souchon et al. [18]	Primary	1	NR	2	NR	NR	MRI	NR	Organ confined	NR	NR	NR	2c
Moore et al. [19]	Primary	1	No	6	Median: 66 (range: 61–71)	TRUS biopsy	MRI Bone scan	NR	Organ confined	≤15	≤3 + 3	NR	2c
Bahn et al. [20]	Primary	2a	NR	31	Mean: 63 (range or SD: NR)	TRUS sextant biopsy plus target biopsy of suspicious areas	TRUS Doppler evaluation	ASTRO	Unilateral	NR	NR	NR	4
Onik et al. [21]	Primary	2a	25 (45%) received short-term ADT (stopped at treatment)	55	NR	TRUS 10-core biopsy or transperineal template biopsy	NR	ASTRO	NR	NR	NR	NR	4
Ellis et al. [22]	Primary	2a	NR	60	Mean: 69 (SD: 7.8)	NR	NR	ASTRO	NR	NR	NR	NR	4
Muto et al. [23]	Primary	2a	7 (24.1%) received short-term ADT (stopped at treatment)	29	Median: 72 (range: 62–80)	TRUS >12-core biopsy	MRI	ASTRO	Unilateral	NR	NR	NR	3b
Murat et al. [24]	Primary	2a	NR	56	Mean: 65.6 (range or SD: NR)	NR	NR	Phoenix	Unilateral	NR	NR	Low-intermediate	4
Lindner et al. [25]	Primary	1	No	12	Median: 56.5 (range: 51–62)	TRUS 12-core biopsy	MRI	NR	Tumour located in 1 of 12 core biopsy sectors	<10	≤3 + 3	Low	2c
Lindner et al. [26]	Primary	1	NR	4	Median: 66 (range: 61–73)	NR	NR	NR	NR	NR	NR	NR	4
Raz et al. [27]	Primary	1	NR	2	73	NR	MRI	NR	NR	NR	NR	NR	4
Truesdale et al. [28]	Primary	2b	No	77	Mean: 69.5 (SD: 6.7)	TRUS biopsy	CT Bone scan	Phoenix	Unilateral	NR	NR	NR	4
El Fegoun et al. [29]	Primary	2a	No	12	Mean: 70 (SD: 4.8)	NR	CT Bone scan	Phoenix	Unilateral	≤10	≤3 + 4	Low-intermediate	4
Ahmed et al. [30]	Primary	2a	No	20	Mean: 60.4 (SD: 5.4)	Transperineal template biopsy	MRI	NR	Unilateral	≤15	≤4 + 3	Low-intermediate	2b
Ward et al. [31]	Primary	2b	NR	1160	Mean: 67.8 (SD: 7.8)	NR	NR	ASTRO	No restriction	No restriction	No restriction	No restriction	4
Tay et al. [32]	Primary	1	No	9	NR	NR	MRI	NR	NR	<10	≤3 + 3	Low	4
Chopra et al. [33]	Primary	1	No	8	Mean: 60 (range: 49–70)	TRUS 12-core biopsy	MRI	NR	NR	≤15	≤4 + 3	Low-intermediate	2c
Bahn et al. [34]*	Primary	2b	13 (18%) received short-term ADT (stopped at treatment)	73	Median: 64 (range: 47–79)	TRUS sextant biopsy plus mapping target biopsy of suspicious areas	TRUS Doppler evaluation	NR	Unilateral	≤20	≤4 + 3	Low-intermediate	4
Ahmed et al. [35]	Primary	2a	No	41	Median: 63 (range: 58–66)	Transperineal template biopsy	MRI	NR	Unilateral and bilateral	≤15	≤4 + 3	Low-intermediate	2b
Dickinson et al. [36]*	Primary	2a	No	88	Median: 64 (range: 48–75)	Transperineal template biopsy	MRI	Phoenix and Stuttgart	Unilateral and bilateral	<20	≤4 + 3	Low-intermediate	2b
Nguyen et al. [37]	Primary	2b	No	318	NR	TRUS biopsy (sextant between 1997–2003, then 10–12 cores)	MRI	Phoenix (and Phoenix plus PSAV >0.75/yr)	No tumour beyond peripheral zone	< 15	≤3 + 4	Low-intermediate	4
Napoli et al. [38]	Primary	1	No	5	Median: 65.4 (range: 50–75)	NR	MRI	NR	Unilateral and unifocal	NR	≤4+ 3	Low-intermediate	2c
Barret et al. [39]	Primary	2b	No	106	Mean: 66.5 (IQR: 61–73)	Transperineal template biopsy (97%) and TRUS 12-core biopsy (100%)	MRI	NR	Unilateral	<10	≤3 + 3	Low	4

bDFS = biochemical disease-free survival; PSA = prostate-specific antigen; NCCN = National Comprehensive Cancer Network; NR = not reported; SD = standard deviation; TRUS = transrectal ultrasound; MRI = magnetic resonance imaging; ASTRO = American Society for Therapeutic Radiology and Oncology; CT = computed tomography; ADT = androgen-deprivation therapy; PSAV = prostate-specific antigen velocity; IQR = interquartile range.

* This series partially overlaps with one previously reported.

**Table 2:** Target population with stage of evaluation and level of evidence of the five studies using focal therapy in the primary setting

									Eligibility criteria				
Reference	Setting	IDEAL stage of evaluation	Previous treatment, no. (%)	Patients, no.	Age, yr	Preoperative biopsy	Preoperative imaging	Criteria for bDFS	Spatial location	PSA level, ng/ml	Gleason score	Risk classification (D’Amico or NCCN)	Level of evidence
Shariat et al. [40]	Salvage (*n* = 8) Primary (*n* = 3)	1	EBRT: 8 (73)	11	Median: 77 (range: 60–82)	TRUS 12-core biopsy	NR	NR	NR	NR	NR	NR	2c
Nguyen et al. [41]	Salvage	2a	EBRT: 12 (48) Brachytherapy: 12 (48) Brachytherapy plus EBRT: 1 (4)	25	Median: 65 (range: 56–82)	TRUS biopsy	CT or MRI Bone scan	Phoenix	NR	<10	≤4 + 3	NR	2b
Eisenberg et al. [42]	Salvage	2a	EBRT with some men (*n* = NR) receiving permanent seed implantation and/or ADT: 18 (100)	18	Mean: 70.6 (range: 58–86)	TRUS 16-core biopsy	CT Bone scan	ASTRO and Phoenix	Unilateral	NR	NR	NR	4
Ahmed et al. [43]	Salvage	2b	EBRT: 39 (100) Short-term ADT (stopped at treatment): 13 (33)	39	Mean: 70.5 (SD: 6.8)	Transperineal template biopsy (*n* = 20) or TRUS biopsy (*n* = 19)	MRI FDG-PET	Phoenix and Stuttgart	NR	NR	NR	NR	4
Abreu et al. [44]	Salvage	2b	EBRT: 11 (44) Proton beam radiotherapy: 8 (32) Brachytheraphy: 5 (20) Brachytheraphy plus EBRT: 1 (4) Short-term ADT (stopped at treatment): 9 (36)	25	Median: 71 (range: 59–81)	TRUS sextant biopsy plus mapping target biopsy of suspicious areas	TRUS Doppler	Phoenix	Unilateral	≤20	No restriction	No restriction	3b

bDFS = biochemical disease-free survival; PSA = prostate-specific antigen; NCCN = National Comprehensive Cancer Network; EBRT = external beam radiation therapy; TRUS = transrectal ultrasound; NR = not reported; CT = computed tomography; MRI = magnetic resonance imaging; ADT = androgen-deprivation therapy; ASTRO = American Society for Therapeutic Radiology and Oncology; FDG-PET = 2-deoxy-2-[F-18]fluoro-D-glucose positron emission tomography.

Twenty-five series in total were identified that evaluated focal therapy in the primary setting (Table 1) [15], [16], [17], [18], [19], [20], [21], [22], [23], [24], [25], [26], [27], [28], [29], [30], [31], [32], [33], [34], [35], [36], [37], [38], [39]. This equates to 2232 men treated using focal therapy and reported in the literature. Six series used cryosurgery, 12 used high-intensity focused ultrasound (HIFU), 1 used photodynamic therapy (PDT), 3 used photothermal therapy, 1 used radiofrequency interstitial tumour ablation (RITA), 1 used magnetic resonance imaging (MRI)-guided brachytherapy, and 1 incorporated various ablation techniques. Median follow-up periods for the reported focal therapy series are 0–10.6 yr (overall range: 0–11.1 yr).

Table 2 summarises the eligibility criteria for patients to be included in the five studies evaluating focal salvage therapy in patients with biochemical failure after radical whole-gland radiotherapy [40], [41], [42], [43], [44]. Apart from one feasibility study investigating the role of RITA in a mixed population of primary and salvage cases, cryoablation, HIFU, and MRI-guided brachytherapy have all been evaluated in a focal manner in patients after external-beam radiation therapy (EBRT) and/or brachytherapy or after proton-beam radiotherapy failure. The number of patients treated in this manner across the series was 115, with a median follow-up of 17–47 mo (range: 3–90 mo).

Thirteen registered trials are evaluating patients treated by focal ablation, with an expected accrual of 989 men [45], [46], [47], [48], [49], [50], [51], [52], [53], [54], [55], [56], [57]. These trials are using cryosurgery (three trials), HIFU (three trials), PDT (three trials), irreversible electroporation (one trial), MRI-guided thermal therapy (one trial), brachytherapy (one trial), and high dose-rate (HDR) brachytherapy for external beam radiotherapy failure (one trial) (Table 3).

**Table 3:** The design of the ongoing 13 registered trials investigating focal therapy using various sources of energy

						Eligibility criteria								
Reference	Leading centre	Trial number	Estimated enrolment, no.	Setting	Technology	Spatial location	PSA level, ng/ml	Gleason score	Risk classification (D’Amico or NCCN)	Follow-up, mo	Primary outcome (measure)	Secondary outcome (measure)	Stage	Status (on registration system)
Eggener [45]	University of Chicago	NCT01192438	9	Primary	MRI-guided laser-induced thermal therapy	NR	NR	≤4 + 3	Low-intermediate	6	Safety	NR	2a	Completed (NR)
Taneja [46]	NYU Urology Associates (multicentre)	NCT00946881	30	Primary	PDT	Unilateral tumour	<10	3 + 3	Low	12	Safety	- Cancer control (biopsy) - QOL outcome	2a	Completed (NR)
Emberton [47]	University College London Hospitals (multicentre)	NCT00975429	86	Primary	PDT	NR	<10	≤3 + 4	Low-intermediate	6	Cancer control (biopsy)	- Urinary outcome (IPSS) - Erectile outcome (IIEF) - QOL outcome	2b	Completed (NR)
Emberton [48]	University College London Hospitals (multicentre)	NCT01310894	200	Primary	PDT	Unilateral tumour	≤10	3 + 3	Low	24	Cancer control (biopsy)	- Urinary outcome - Erectile outcome - QOL outcome	2b	Recruiting
Emberton [49]	University College London Hospitals (multicentre)	NCT01194648	272	Primary	HIFU	Unilateral clinically significant disease	≤15	≤4 + 3	Low-intermediate	36	Cancer control (biopsy)	- Urinary outcome (IPSS and UCLA-EPIC urinary domain) - Erectile outcome (IIEF-15) - Rectal outcome (UCLA-EPIC bowel domain) - QOL (EQ-5D and RAND 36-item Health Survey125)	2b	Recruiting
Ahmed [50]	University College London Hospitals	NCT00987675	56	Primary	HIFU	Any localisation, but preservation of at least one neurovascular bundle	<20	≤8	All risks	12	Safety	- Cancer control (biopsy and PSA kinetics) - Urinary outcome (IPSS and UCLA-EPIC urinary domain) - Erectile outcome (IIEF-15) - Rectal outcome (UCLA-EPIC bowel domain) - QOL (FACT-P)	2a	Recruiting
Guazzoni [51]	Università Vita-Salute San Raffaele	NCT00928603	100	Primary	Cryoablation	Tumours in the transition zone are excluded	<10	3 + 3	Low	60	Safety	- Cancer control - Urinary outcome (IPSS) - Erectile outcome (IIEF-15) - QOL (FACT-P and MSKCC Prostate-Health Related Quality of Life Questionnaire)	2b	Recruiting
Napoli [52]	University of Roma La Sapienza	NCT01522118	12	Primary	MRI-guided HIFU	NR	≤10	≤3 + 4	Low-intermediate	18	Safety	- Cancer control (biopsy)	2a	Recruiting
Zelefsky [53]	Memorial Sloan-Kettering Cancer Centre	NCT01354951	80	Primary	Brachytherapy	Unilateral tumour	<10	≤3 + 4	Low-intermediate	24	Safety (NCI CTCAE)	- Cancer control (biopsy) - QOL (MSKCC Prostate-Health Related Quality of Life Questionnaire)	2b	Recruiting
Ward [54]	UT MD Anderson Cancer Centre	NCT00877682	100	Primary	Cryoablation	NR	≤10	≤3 + 4	Low-intermediate	36	Cancer control (biopsy)	- Urinary outcome - Erectile outcome - Rectal outcome - QOL outcome	2b	Recruiting
Eastham [55]	Memorial Sloan-Kettering Cancer Centre	NCT00774436	50	Primary	Cryoablation	NR	<10	NR	Low	6	Cancer control (biopsy)	- QOL	2b	Not yet recruiting
Emberton [56]	University College London Hospitals	NCT01726894	20	Primary	Irreversible electroporation	Anterior tumour	≤15	≤4 + 3	Low-intermediate	12	Safety (NCI CTCAE)	- Cancer control (biopsy) - Urinary outcome (IPSS and UCLA-EPIC urinary domain) - Erectile outcome (IIEF-15) - Rectal outcome (UCLA-EPIC bowel domain) - QOL (EQ-5D, FACT-P and Memorial Anxiety scale)	2a	Not yet recruiting
Chung [57]	Sunnybrook Health Sciences Centre, Odette Cancer Centre	NCT01583920	4	Salvage	HDR brachytherapy	Recurrence confined to the prostate	<10 ≤20 (before EBRT)	≤ 4 + 3 (before EBRT)	Low-intermediate	60	Safety (NCI CTCAE)	- Cancer control (bDFS) - Urinary outcome (IPSS) - QOL (UCLA-EPIC)	2a	Not yet recruiting

PSA = prostate-specific antigen; NCCN = National Comprehensive Cancer Network; MRI = magnetic resonance imaging; NR = not reported; PDT = photodynamic therapy; QOL = quality of life; IPSS = International Prostate Symptom Score; IIEF = International Index of Erectile Function; HIFU = high-intensity focused ultrasound; UCLA-EPIC = University of California, Los Angeles-Expanded Prostate Cancer Index Composite; EQ-5D = EuroQol-5 dimensions; RAND 36 = RAND 36-item health survey; FACT-P = Functional Assessment of Cancer Therapy-Prostate; NCI CTCAE = National Cancer Institute Common Terminology Criteria for Adverse Events; MSKCC = Memorial Sloan-Kettering Cancer Center; HDR = high dose rate; bDFS = biochemical disease-free survival.

### 3.2 Defining the ideal candidate for focal therapy

No overall consensus exists for defining the ideal candidate for primary focal therapy, despite several consensus statements. This reflects different schools of thought with respect to the role of focal therapy in PCa. In 2007, the International Task Force on PCa proposed very conservative criteria for selecting patients, essentially deeming focal therapy an alternative to active surveillance in very low-risk disease [58]. These criteria were a PSA level <10 ng/ml, the absence of Gleason grade 4 and 5, the use of extended biopsy schemes, and very restricted biopsy criteria, including maximum length of cancer in each core of 7 mm and maximum percentage of total cores with cancer of 33%. Other consensus groups have attempted to introduce greater flexibility in these criteria by essentially allowing intermediate-risk and some higher-risk PCas, effectively deeming focal therapy an alternative strategy for those men who would normally be advised to have radical therapy [7], [59], [60], [61].

The criteria used to select candidates for focal therapy in the primary setting do not reflect the conservative approach initially laid down in 2007, and they show a predilection towards intermediate-risk cancer as well as low-risk disease (Table 1). Most studies have excluded patients with very low-risk disease and recruited men with presumed unilateral disease. In summary, 1109 men with low-risk disease (56%), 704 men with intermediate-risk disease (36%), and 164 men with high-risk disease (8%) were treated with focal therapy [15], [16], [17], [18], [19], [20], [21], [22], [23], [24], [25], [26], [27], [28], [29], [30], [31], [32], [33], [34], [35], [36], [37], [38], [39]. Risk categories were not available in 13 series. The PSA level was 3.76–24 ng/ml (overall range: 0.01–82.2 ng/ml), and median age ranged from 56.5 to 73 yr (overall range: 47–80 yr) among the studies. Individual Gleason attribution was available in 20 series, with 1503 men with Gleason score ≤6, 521 with Gleason score 7, and 82 men with Gleason score ≥8.

In focal salvage series, patients were older, with the median age ranging from 65 to 77 yr. Eighty-eight treated men (76%) had failure following EBRT, 17 (15%) after brachytherapy, 2 (2%) after brachytherapy combined with EBRT, and 8 (7%) after proton beam therapy (Table 2). No restriction in PSA value or initial risk classification was applied in most studies. However, two series included only patients with presumed unilateral disease [42], [44].

Of the ongoing trials in the primary setting, four are recruiting only low-risk disease, seven are recruiting low- through intermediate-risk disease, and one has no risk restriction (Table 3). Finally, one trial is evaluating focal HDR brachytherapy after EBRT failure.

### 3.3 Disease localisation

The spatial location of the tumour within the prostate is essential for focal therapy to deliver treatment. There is no accepted standard for disease localisation for the purpose of delivering focal therapy. The consensus group statements in this area have made recommendations based on limited information at the time of writing. Most have recommended either extended transrectal ultrasound (TRUS) biopsies with strict low-risk criteria or the use of adjunctive imaging, usually multiparametric MRI (mpMRI). Studies have shown that TRUS biopsy is inaccurate for the purposes of identifying candidates for focal therapy and for localising disease [62], [63], [64], [65], [66], [67], [68], [69], [70]. As a result, most consensus statements have recommended that transperineal template mapping biopsies (TTMBs) are the gold standard for disease localisation for the purpose of focal therapy while accepting that this procedure is more invasive and has health-care resource implications, although the risk of sepsis is negligible [71], [72], [73], [74], [75], [76], [77], [78], [79]. In addition, there is no consensus as to how many biopsies are sufficient to detect all or most clinically significant cancer. A recent study has shown that TTMB using a 5-mm sampling frame missed only one lesion from a total of 64 lesions that had a volume of ≥0.5 ml and/or had elements of Gleason pattern 4 on subsequent whole-mount radical prostatectomy specimens [72].

Imaging in the form of a high-quality mpMRI reported by expert radiologists may have the performance characteristics required to localise significant areas of PCa. Evidence is building to show that an area deemed negative on mpMRI stands a 95% probability of having no clinically significant disease as defined by the presence of any Gleason pattern 4 and/or a lesion volume of ≥0.5 ml [80], [81]. Other ultrasound modalities are demonstrating promise but presently lack the weight of evidence for mpMRI [82], [83], [84], [85].

In our systematic review, most of the studies used some form of preoperative MRI in combination with biopsy parameters as criteria to select patients; some recent series use this modality for treatment planning (Table 4, Table 5) [27], [32], [33], [37], [38], [41]. In summary, among the primary selected studies, two series used only TRUS biopsy, two used TRUS biopsy and Doppler ultrasound, six used TRUS biopsy and MRI, and four used TTMB and mpMRI. The preoperative assessment was not reported in 11 studies.

**Table 4:** Actual population studied in each primary series with the histologic, biochemical, and cancer long-term outcomes

Reference	Technology	Type of ablation	PSA level, ng/ml	Gleason score at preoperative biopsy (%)	Risk classification, D’Amico or NCCN, no. (%)	Follow-up	Postfocal histology (reason)	Presence of any cancer, actual (%)	Presence of clinically significant cancer	bDFS, %	PSA kinetics*	Secondary treatment, actual (%)	Metastatic disease, actual (%)	Mortality: %
Madersbacher et al. [15]	HIFU	Midline target or unilateral ablation of TRUS-visible tumours	Mean: 24 (range: 2–82.8)	NR	NR	Few hours (mean/median: NR)	RP	29/29 (100)	NR	NR	NR	NR	NR	Overall and cancer-specific survival: 100
Zlotta et al. [16]	RITA	Multifocal	NR	NR	NR	Mean/median: NR (range: 0 d–3 mo)	RP	14/14 (100)	NR	NR	NR	NR	NR	Overall and cancer-specific survival: 100
Beerlage et al. [17]	HIFU	Total or subtotal hemiablation	Mean: 10.8 (range: 3.5–20)	NR	NR	Median: 8.5 d (range: 7–12)	RP	13/14 (93) 4/14 (29) had residual tumor in treated area	NR	NR	NR	0/14 (0)	0/14 (0)	Overall and cancer-specific survival: 100
Souchon et al. [18]	HIFU	Focal ablation of peripheral zone	NR	NR	NR	NR	NR	NR	NR	NR	NR	NR	NR	NR
Moore et al. [19]	PDT	Focal with ipsilateral peripheral zone ablation	Median: 6.95 (range: 1.9–15)	3 + 3: 6 (100)	NR	NR	TRUS sextant biopsy (protocol)	6/6 (100)	NR	NR	NR	4/6 (67) redo PDT 5/6 (83) salvage treatment (3 EBRT, 1 brachytherapy, 1 cryotherapy)	0/6 (0)	Overall and cancer-specific survival: 100
Bahn et al. [20]	Cryoablation	Hemiablation	Mean: 4.95 (range or SD: NR)	≤6: 23 (74) 7: 8 (26)	NR	Mean: 70 mo (range: 2–107)	TRUS sextant biopsy plus target biopsy of suspicious areas on Doppler (protocol)	1/25 (4)	NR	92.9	NR	1/31 (4) redo cryoablation	0/25 (0)	Overall survival: 96 Cancer-specific survival: 100
Onik et al. [21]	Cryoablation	Focal	Mean: 8.3 (range or SD: NR)	NR	Low: 26 (48) Intermediate: 20 (36) High: 9 (16)	Mean: 3.6 yr (range: 1–10)	NR	Only patients having biopsy: 4/30 (13) All patients: 4/55 (7)	NR	95 (3 yr)	Mean: 2.4 (SD: NR)	4/55 (7) redo cryoablation	NR	Overall and cancer-specific survival: 100
Ellis et al. [22]	Cryoablation	Hemiablation plus contralateral peripheral zone (hockey stick)	Mean: 7.2 (SD: 4.7)	≤6: NR (78.3) 7: NR (20) ≥8 NR (1.7)	Low: 40 (66.7) Intermediate: 14 (23.3) High: 6 (10)	Median: 12 mo (range: 3–36)	NR	Only patients having biopsy: 14/35 (40); 1/35 (3) in treated side All patients: 14/60 (23); 1/60 (1.7) in treated side	NR	80.4	Median: 1.7 (IQR: NR)	11/60 (18) redo cryoablation	0/60 (0)	Overall and cancer-specific survival: 100
Muto et al. [23]	HIFU	Hemiablation plus contralateral peripheral zone (hockey stick)	Median: 5.4 (range: 0.2–25.1)	Unknown: 2 (6.9) ≤6: 16 (55.2) 7: 6 (20.7) ≥8: 5 (17.2)	NR	Median: 34 mo (range: 8–45)	TRUS sextant (protocol)	At 6 mo: 3/28 (10.7) At 12 mo: 4/17 (23.5)	NR	2-yr Low risk: 83.3 Intermediate risk: 53.6	36-mo mean: 1.89 (SD: 1.51)	7/29 (24) ADT	NR	Overall and cancer-specific survival: 100
Murat et al. [24]	HIFU	Hemiablation	NR	NR	Low: 33 (59) Intermediate: 23 (41)	Median: 42 mo (NR)	NR	NR	NR	3 yr: 76 5 yr: 60	Nadir after first HIFU: mean: 0.5 (SD: NR) Nadir after redo HIFU: mean: 0.47 (SD: NR)	19/56 (34) redo HIFU	NR	NR
Lindner et al. [25]	Photothermal laser	Focal	Mean: 5.7 (SD: 1.1)	3 + 3: 12 (100)	Low risk: 12 (100)	6 mo	TRUS 10-core biopsy plus 2 cores guided in the treated area (protocol)	6/12 (50) 4/12 (33) in treated area	2/12 (17)	NR	NR	1/12 (8) RP	0/12 (0)	Overall and cancer-specific survival: 100
Lindner et al. [26]	Photothermal laser	Focal	Median: 4.2 (range: 2.9–14.8)	3 + 3: 2 (50) 4 + 3: 2 (50)	NR	1 wk	RP	4/4 (100) with no residual tumor in treated area	NR	NR	NR	NR	NR	Overall and cancer-specific survival: 100
Raz et al. [27]	Photothermal laser	Focal	Median: 3.76 (range: 2.74–4.79)	3 + 3: 2 (100)	Low: 2 (100)	≤1 mo	NR	NR	NR	NR	NR	0/2 (0)	NR	Overall and cancer-specific survival: 100
Truesdale et al. [28]	Cryoablation	Hemiablation	Mean: 6.54 (SD: 4.87)	≤6: 50 (65) 7: 25 (32) 8: 2 (3)	Low: 44 (57) Intermediate: 31 (40) High: 2 (3)	Median: 24 mo (0–87)	TRUS 12-core biopsy (for cause)	Only patients having biopsy: 10/22 (45.5) 3/22 (14) in treated area All patients: 10/77 (13); 3/77 (3.9) in treated area	NR	72.7	Mean: 1.23 (SD: 1.38)	NR	NR	Overall and cancer-specific survival: 100
El Fegoun et al. [29]	HIFU	Hemiablation	Mean: 7.3 (range: 2.6–10)	≤3 + 3: 10 (83) 3 + 4: 2 (17)	NR	Median: 10.6 yr (range: 7.5–11.1)	TRUS sextant (protocol)	1/12 (8)	0/12	5 yr: 90 10 yr: 38	Median: 1.5 (range: 0.1–6.8)	1/12 (8) redo HIFU 4/12 (33) ADT	0/12 (0)	Overall survival: 83 Cancer-specific survival: 100
Ahmed et al. [30]	HIFU	Hemiablation	Median: 7.3 (range: 3.4–11.8)	NR	Low: 5 (25) Intermediate: 15 (75)	12 mo	TRUS biopsy of the treated side (protocol)	2/19 (11)	0/19	NR	12-mo mean: 1.5 (SD: 1.3)	1/20 (5) redo HIFU	0/19 (0)	Overall and cancer-specific survival: 100
Ward et al. [31]	Cryoablation	NR	1149 (99%) available <4: 211 (18%) 4 to <10: 782 (68%) 10 to <20: 126 (11%) >20: 30 (3%)	1148 (99) available ≤6: 844 (74) 7: 240 (21) ≥8: 64 (5)	1157 (99) available Low: 541 (47) Intermediate: 473 (41) High: 143 (12)	Mean: 21.1 mo (SD: 19.7)	NR	Only patients having biopsy: 43/163 (26.3) All patients: 43/1160 (3.7)	NR	3 yr: 75.7	NR	NR	NR	NR
Tay et al. [32]	MRI-guided HIFU	Uni- or multifocal	NR	NR	NR	NR	NR	0/1	NR	NR	NR	NR	NR	NR
Chopra et al. [33]	MRI-guided HIFU	Peripheral zone	Mean: 6.2 (range: 2.7–13.1)	3 + 3: 2 (25) 3 + 4: 4 (50) 4 + 3: 2 (25)	NR	<2 h	RP	8/8 (100)	6/8 (75)	NR	NR	NR	0/8 (0)	Overall and cancer-specific survival: 100
Bahn et al. [34]**	Cryoablation	Hemiablation	Median: 5.4 (range: 0.01–20)	3 + 3: 30 (41) 3 + 4: 25 (34) 4 + 3: 18 (25)	Low: 24 (33) Intermediate: 49 (67)	Median: 3.7 yr (range: 1–8.5)	TRUS sextant plus target biopsy of suspicious areas (protocol)	12/48 (25) 11 positive biopsy results in untreated side; 1 treated side	5/48 (10)	NR	36-mo mean: 2.1 (SD: 3.8)	2/73 (3) redo cryoablation 1/73 (1.4) brachytherapy, 1/73 (1.4) ADT	0/48 (0)	Overall and cancer-specific survival: 100
Ahmed et al. [35]	HIFU	Unilateral, bilateral or midline ablation	Median: 6.6 (range: 5.4–7.7)	3 + 3: 13 (32) 3 + 4: 24 (59) 4 + 3: 4 (10)	Low: 11 (27) Intermediate: 26 (63) High: 4 (10)	12 mo	TRUS biopsy of treated area (protocol)	9/39 (23)	3/39 (8)	NR	Median: 1.9 (IQR: 0.8–3.3)	4/41 (10) redo HIFU	0/39 (0)	Overall survival: 97.7 Cancer-specific survival: 100
Dickinson et al. [36]**	HIFU	Hemiablation, unifocal or multifocal	NR	3 + 3: 31 (35) 3 + 4: 50 (57) 4 + 3: 7 (8)	NR	Median: 32 mo (range: 24–69)	TRUS biopsy of treated area (protocol)	20/72 (28)	10/72 (14)	Phoenix 71/87 (82) Stuttgart 57/87 (66)	NR	NR/87 (20) redo HIFU 4/87 (5) salvage EBRT	NR	NR
Nguyen et al. [37]	MRI-guided brachytherapy	Peripheral zone	Median: 5.0 (IQR: 3.8–6.9)	3 + 3: 280 (88) 3 + 4: 38 (12)	Low: 265 (83) Intermediate: 53 (17)	5.1 yr (IQR: 2.8–7.3)	TRUS 12-core biopsy (for cause)	Only patients having biopsy: 17/24 (71) All patients: 17/318 (5.3)	NR	Phoenix: 5 yr: 91.5 8 yr: 78.1 Phoenix and PSAV: >0.75/yr 5 yr: 91.9 8 yr: 86.2	NR	NR	1/318 (0.3)	Overall and cancer-specific survival: 99.7
Napoli et al. [38]	MRI-guided HIFU	Focal	Median: 8.8	3 + 3: 3 (60) 3 + 4: 2 (40)	NR	Mean: 9 mo (range: 7–14)	RP	5/5 (100)	NR	NR	NR	NR	NR	NR
Barret et al. [39]	HIFU: 21 (20) Brachytherapy: 12 (11) Cryoablation: 50 (47) PDT: 23 (22)	Hemiablation	Mean: 6.1 (IQR: 5–8.1)	3 + 3: 106 (100)	Low: 106 (100)	Median: 9 mo (range: 6–15)	NR	NR	NR	NR	12-mo median: 2.7 (IQR: 1–4.4)	NR	NR	NR

PSA = prostate-specific antigen; NCCN = National Comprehensive Cancer Network; bDFS = biochemical disease-free survival; HIFU = high intensity focused ultrasound; TRUS = transrectal ultrasound; NR = not reported; RP = radical prostatectomy; RITA = radiofrequency interstitial tumor ablation; PDT = photodynamic therapy; EBRT = external beam radiotherapy; SD = standard deviation; IQR = interquartile range; ADT = androgen-deprivation therapy; MRI = magnetic resonance imaging; PSAV = prostate-specific antigen velocity.

* At last follow-up unless otherwise stated.

** This series partially overlaps with one previously reported.

**Table 5:** Actual population studied in each salvage series with the histologic, biochemical, and cancer long-term outcomes

Reference	Technology	Type of ablation	PSA level, ng/ml	Gleason score at preoperative biopsy (%)	Risk classification (D’Amico or NCCN)	Follow-up, mo, median no. (range)	Postfocal histology (reason)	Presence of any cancer	Presence of clinically significant cancer, actual (%)	bDFS, %	PSA kinetics (at last follow-up unless otherwise stated)	Secondary treatment, actual (%)	Metastatic disease, actual (%)	Mortality, %
Shariat et al. [40]	RITA	Focal	Median: 5.7 (range: 0.66–10.8)	Median: 7; range: 6–8	NA	20 (3–38)	TRUS 12-core biopsy (protocol)	At 12 mo: 3/6 (50), 2/6 (33) in the treated area	NR	NR	90% experienced a decrease in PSA >50% (discrete values: NR)	NR	NR	Overall and cancer-specific survival: 100
Nguyen et al. [41]	MRI-guided brachytherapy	Peripheral zone	Median: 5.5 (range: 1.4–11.6)	2+3: 1 (4) 3+3: 18 (72) 3+4: 6 (24)	NA	47 (14–75)	NR	NR	NR	4 yr: 70	NR	NR	NR	NR
Eisenberg et al. [42]	Cryoablation	Hemiablation	Median: 3.3 (range: 0.28–8.96)	NR	NA	18 (6–33)	TRUS biopsy (protocol)	At 12 mo: 1/10 (10) overall and in the treated area	NR	ASTRO 1 yr: 89 2 yr: 67 3 yr: 50 Phoenix 1 yr: 89 2 yr: 79 3 yr: 79	NR	NR	3/15 (20)	NR
Ahmed et al. [43]	HIFU	Hemiablation (*n* = 16) or quadrant (*n* = 23)	Median: 3.3 (range: 0.02–27.9)	Unknown: 1 (3) 6: 2 (5) 7: 32 (82) ≥8: −4 (10)	NA	17 (10–29)	Transperineal template biopsy (for cause)	Only patients having biopsy: 4/9 (44) All patients: 4/39 (10)	NR	2 yr: Phoenix: 49 Stuttgart: 42	Median: 0.57 (IQR: 0.1–2.3)	16/39 (41) had ADT	2/39 (5)	NR
Abreu et al. [44]	Cryoablation	Hemiablation	Median: 2.8 (range: 0–8.2)	≤3 + 3: 5 (20) 3 + 4: 6 (24) 4 + 3: 8 (32) 4 + 4: 6 (24)	NA	31 (4–90)	TRUS sextant plus mapping target biopsy of suspicious areas (protocol)	2/25 (8)	2/25 (8)	5 yr: 54	At 36 mo: mean: 1.2 (SD: 1.6)	2/25 (8)	No	Overall and cancer-specific survival: 100

PSA = prostate-specific antigen; NCCN = National Comprehensive Cancer Network; bDFS = biochemical disease-free survival; RITA = radiofrequency interstitial tumour ablation; NA = not applicable; NR = not reported; TRUS = transrectal ultrasound; MRI = magnetic resonance imaging; ASTRO = American Society for Therapeutic Radiology and Oncology; HIFU = high-intensity focused ultrasound.

### 3.4 Identifying which lesions to target

PCa, as it is currently defined, is multifocal in about 80% of cases on whole-mount pathology, especially if a finer sampling frame of 3 mm is used [86]. This has generally been regarded as a major limitation in the whole rationale for focal therapy in PCa. Several areas of evidence suggest that multifocality is not necessarily a limiting factor for tissue preservation. First, multifocal disease is present in many other cancers in which tissue-preserving therapy is now standard care [87], [88], [89], [90]. Second, for PCa, unilateral disease is present in up to one-third of men who have surgery [70], [91], [92], [93], [94], [95], [96], [97]. Third, there has been increasing debate and gradual acceptance that not all tumours in the prostate behave similarly. The index-lesion concept proposes that it is only the dominant lesion that drives the natural history of the disease [98], [99], [100], [101]. Indeed, this concept has been extended further by stating that some lesions are clinically significant (likely to have an impact on quality and longevity of life), whereas others are clinically insignificant [10], [102], [103], [104], [105], [106], [107], [108], [109]. Men who have only clinically insignificant disease have little to no chance of disease progression within their lifetime, and some have proposed they would have no certain benefit in being treated with active therapies [110].

Current trials have differed in the approach to ablative strategies. Most investigators aim to treat all known areas of cancer in a hemiablative fashion once a man's PCa is deemed unilateral. Some trials have deliberately allowed for ablation of the index lesion alone even when multifocal disease is found [49], [50], [56]. In reality, it is likely that the difference among these trials is very small, since the studies leaving behind untreated cancer for surveillance use biopsy strategies, such as TTMB with 5-mm sampling, that have a high sampling density. Consequently, in these studies, small lesions, which are likely to be missed by other less accurate sampling strategies, are located but are deliberately left untreated.

In summary, all reported series have treated all known areas of cancer, and no reported series have explicitly stated that therapy was aimed at the index lesion and that lesions were deliberately left untreated. Most ongoing trials aim to treat all known areas of cancer, although three trials are explicitly aiming treatment at the index or at clinically significant lesions with surveillance of untreated insignificant lesions (Table 3).

**Fig. 2:**
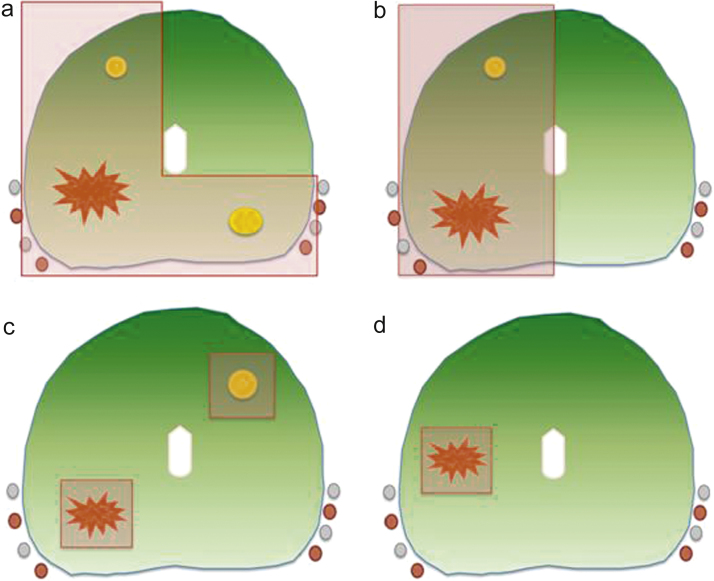
Different tissue-preserving strategies have been used across different series: (a) hockey stick, (b) hemiablation, (c) multifocal, and (d) unifocal strategies are shown in this representative scheme.

In the largest series of 1160 men using cryoablation and in another series using HIFU with multiple strategies (*n* = 88), it was not possible to determine the extent of tissue ablation per patient [31], [36]. Either hemiablation or focal ablation was used in the remaining studies: 12 used a hemiablation or an extended “dog leg” or hockey stick approach (*n* = 537; relative percentage: 49%), 16 used focal/zonal ablation (*n* = 562; relative percentage: 51%), and 3 used bilateral focal ablation when multifocal disease was present (*n* = 65; relative percentage: 6%) (Fig. 2).

### 3.5 Defining success and failure after focal therapy

Another major challenge in focal therapy is the definition of what constitutes success and failure. The use of disease-specific and overall mortality would require large-scale RCTs, which would likely take 5 yr to recruit and then 10–15 yr of follow-up to obtain sufficient event rates to prove noninferiority over radical whole-gland therapies or superiority over active surveillance. As a surrogate, although PSA outcomes are accepted as a valid outcome in standard therapies, the clinical utility of PSA kinetics in tissue preservation is yet to be determined. Since no PSA outcome measure has been validated in focal therapy yet, the criteria used for defining radiotherapy failure have been used across most of the studies. Thus, the majority of the investigators have reported biochemical outcomes using Phoenix or American Society for Therapeutic Radiology and Oncology (ASTRO) criteria [111]. However, these criteria are neither validated nor appropriate for ablative techniques, not only because there is prostatic tissue remaining but also because the mechanism of cell death is different between radiation therapy and immediate ablation, so PSA kinetics are likely to be different. Some have proposed using the Stuttgart definition developed for whole-gland HIFU [112]. One study investigated the biochemical disease-free survival (bDFS) predictive value, verified by follow-up biopsy, of Phoenix criteria alone or Phoenix associated with PSA velocity <0.75 ng/ml per year [37]. The authors found that the compound strategy could predict biopsy-proven failure better than Phoenix criteria alone. If PSA kinetics are used to define focal therapy outcomes, it is likely that such models will have to incorporate the fact that untreated tissue is still PSA secreting and a threshold PSA for failure may have to incorporate an estimation of the extent of prostatic tissue ablated. However, until a validated PSA measure is found, an international consensus is needed about what might constitute biochemical failure after focal therapy, so that medium-term outcomes can be used to allow comparison between individual focal-therapy series, and between focal therapy and standard care.

Our systematic review of focal-therapy series demonstrates the summary outcomes presented in the following section and in Table 4, Table 5, Table 6, Table 7. Furthermore, Table 3 shows outcomes used in prospective registered clinical trials that have not yet been reported.

**Table 6:** Perioperative and functional outcome of patients undergoing focal therapy in the primary setting

Reference	Length of stay, d, median	Anaesthesia	Complications, actual (%)	Urinary continence, actual (%)	Erectile function*, actual (%)	Rectal toxicity, actual (%)	Quality of life	Trifecta outcome**, actual (%)
Madersbacher et al. [15]	NR	General	NR	NR	NR	NR	NR	NR
Zlotta et al. [16]	NR	General or spinal	NR	NR	NR	NR	NR	NR
Beerlage et al. [17]	2	General or spinal	NR	NR	NR	Rectourethral fistula: 0/14 (0) Perineal pain: 14/14 (100) Rectal bleeding: NR Diarrhoea: NR PROM: NR	NR	NR
Souchon et al. [18]	NR	NR	NR	NR	NR	NR	NR	NR
Moore et al. [19]	1	General	Urinary retention: 1/6 (17) Urethral stricture: NR UTI: 1/6 (17) Outcome measure: NR	Pad free: NR Leak free: 5/6 (83) PROM: AUA-7	1/3 (33) PROM: Brief Sexual Function Inventory	Rectourethral fistula: 0/3 (0) Perineal pain: NR Rectal bleeding: 2/6 (33) Diarrhoea: 2/6 (33) PROM: NR	NR	NR
Bahn et al. [20]	NR	NR	NR	Pad free: 28/28 (100) Leak free: NR PROM: NR	24/27 (88.8) PROM: Brief Male Sexual Function Index	NR	NR	NR
Onik et al. [21]	NR	NR	NR	Pad free: 24/25 (96) Leak free: NR PROM: NR	44/51 (85) PROM: NR	NR	NR	NR
Ellis et al. [22]	1	NR	NR	Pad free: 55/55 (100) Leak free: 53/55 (96.4) PROM: NR	24/34 (70.6) PROM: NR (vacuum therapy and oral therapy for erectile dysfunction offered preoperatively)	Rectourethral fistula: 0/34 (0) Perineal pain: NR Rectal bleeding: NR Diarrhoea: NR PROM: NR	NR	NR
Muto et al. [23]	1	NR	Urinary retention: NR Urethral stricture 1/25 (4) UTI 1/25(4) Outcome measure: NR	Pad free: NR Leak free: NR PROM: UCLA-EPIC, IPSS	NR	NR	NR	NR
Murat et al. [24]	NR	NR	NR	NR	28/52 (54) PROM: IIEF-5	NR	NR	NR
Lindner et al. [25]	1	General	Urinary retention: 0/12 (0) Urethral stricture: 0/12 (0) UTI: 0/12 (0) Outcome measure: NR	Pad free: 12/12 (100) Leak free: 12/12 (100) PROM: IPSS	NR (100) PROM: IIEF-5	Rectourethral fistula: 0/12 (0) Perineal pain: 3/12 (25) Rectal bleeding: 0/12 (0) Diarrhoea: 0/12 (0) PROM: NR	NR	6/12 (50)
Lindner et al. [26]	NR	NR	NR	NR	NR	NR	NR	NR
Raz et al. [27]	1	General	NR	NR	NR	NR	NR	NR
Truesdale et al. [28]	1	General or spinal	NR	Pad free: 77/77 (100) Leak free: NR PROM: IPSS	NR PROM: IIEF	NR	NR	NR
El Fegoun et al. [29]	NR	NR	Urinary retention: 1/12 (8) Urinary stricture: 0/12 (0) UTI: 2/12 (16) Outcome measure: NR	Pad free: 12/12 (100) Leak free: NR PROM: IPSS	NR	NR	NR	NR
Ahmed et al. [30]	1 day	General	Urinary retention: 0/20 (0) Urinary stricture: 1/20 (5) UTI: 0/20 (05) Outcome measure: NR	Pad free: 19/20 (95) Leak free: 18/20 (90) PROM: UCLA-EPIC, IPSS	19/20 (95) PROM: IIEF-15	Rectourethral fistula: 0/20 (0) Perineal pain: NR Rectal bleeding: NR Diarrhoea: NR PROM: FACT-P	No significant difference between baseline and last follow-up PROM: FACT-P, FACT-G	17/19 (89)
Ward et al. [31]	NR	NR	Urinary retention: 6/518 (1.1) Urinary stricture: NR UTI: NR Outcome measure: NR	Pad free: 499/507 (98.4) Leak free: NR PROM: NR	169/291 (58.1) PROM: NR	Rectourethral fistula: 1/507 (0.1) Perineal pain: NR Rectal bleeding: NR Diarrhoea: NR PROM: NR	NR	NR
Tay et al. [32]	NR	NR	NR	NR	NR	NR	NR	NR
Chopra et al. [33]	NR	Spinal	NR	NR	NR	NR	NR	NR
Bahn et al. [34]†	1	NR	NR	Pad free: 73/73 (100) Leak free: NR PROM: NR	36/42 (86) PROM: IIEF-5	Rectourethral fistula: 0/73 (0) Perineal pain: NR Rectal bleeding: NR Diarrhoea: NR PROM: NR	NR	NR
Ahmed et al. [35]	1	General	Urinary retention: 1/41 (2.4) Urethral stricture: 0/41 (0) UTI: 0/41 (0) Outcome measure: NR	Pad free: 40/40 (100) Leak free: 39/39 (100) PROM: UCLA-EPIC, IPSS	31/35 (86) PROM: IIEF-15	Rectourethral fistula: suspicion in 1/41 (2.4) Perineal pain: NR Rectal bleeding: NR Diarrhoea: NR PROM: NR	Significant small deterioration between baseline and last follow-up PROM: FACT-P, FACT-G	26/31 (84)
Dickinson et al. [36]†	1	General	NR	Pad free: 86/87 (99) Leak free: 56/66 (85) PROM: IPSS, UCLA-EPIC	76/85 (89) PROM: IIEF-15	Rectourethral fistula: 1/88 (1) Perineal pain: NR Rectal bleeding: NR Diarrhoea: NR PROM: NR	Significant deterioration between baseline and last follow-up PROM: FACT-P	NR
Nguyen et al. [37]	1	General	NR	NR	NR	NR	NR	NR
Napoli et al. [38]	1	NR	NR	NR	NR	NR	NR	NR
Barret et al. [39]	1–2	NR	Urinary retention: 9/106 (8) Urethral stricture: 1/106 (1) UTI: 0/106 (0) Outcome measure: Clavien-Dindo classification (13% complication rate, 2% major)	Pad free: 106/106 (100) Leak free: NR PROM: IPSS	NR PROM: IIEF-5	Rectourethral fistula: 1/106 (1) Perineal pain: 1/106 (1) Rectal bleeding: 0 Diarrhoea: NR PROM: NR	NR	NR

NR = not reported; PROM = patient-reported outcome measure; UTI = urinary tract infection; AUA-7 = American Urological Association symptom index 7; UCLA-EPIC = University of California, Los Angeles-Expanded Prostate Cancer Index Composite; IPSS = International Prostate Symptom Score; IIEF = International Index of Erectile Function; FACT-P = Functional Assessment of Cancer Therapy-Prostate; FACT-G = Functional Assessment of Cancer Therapy-General.

* Ability to have penetrative intercourse.

** Pad-free, leak-free continence; erections sufficient for penetration; absence of clinically significant disease after focal therapy.

† This series partially overlaps with one previously reported.

**Table 7:** Perioperative and functional outcome of patients undergoing focal therapy after radiotherapy failure

Reference	Length of stay, d, median	Anaesthesia	Complications, actual (%)	Urinary continence, actual (%)	Erectile function*, actual (%)	Rectal toxicity, actual (%)	Quality of life	Trifecta outcome**
Shariat et al. [40]	1	Sedation	NR	Pad free: NR Leak free: NR PROM: IPSS	NR	NR	Difference between baseline and last follow-up: No PROM: Quality of Life Index	NR
Nguyen et al. [41]	1	General	Urinary retention: NR Urethral stricture: 1/25 (4) UTI: NR Outcome measure: RTOG	Pad free: 22/25 (88%) Leak free: NR PROM: NR	NR	Rectourethral fistula: 3/25 (12) Perineal pain: NR Rectal bleeding: 2/25 (8) Diarrhoea: NR PROM: RTOG	NR	NR
Eisenberg et al. [42]	NR	General or spinal	Urinary retention: 0/15 (0) Urethral stricture: 1/15 (7) UTI: NR Outcome measure: NR	Pad free: 14/15 (93.3) Leak free: NR PROM: NR	Ability to have penetrative sex: 2/5 (40) PROM: Sexual Health Inventory for Men	Rectourethral fistula: suspicion 1/15 (7) Perineal pain: 1/15 (7) Rectal bleeding: NR Diarrhoea: NR PROM: NR	NR	NR
Ahmed et al. [43]	1	General	Urinary retention: NR Urethral stricture: 1/39 (3) UTI: 3/39 (8) Outcome measure: Clavien-Dindo grade; 1: 3 (8), 2: 0, 3a: 1 (3), 3b: 9 (23), 4:0	Pad free: 34/39 (87.2) Leak free: 25/39 (64.1) PROM: UCLA-EPIC, IPSS	Ability to have penetrative sex: NR PROM: IIEF-15	Rectourethral fistula: 1/39 (2.6) Perineal pain: NR Rectal bleeding: NR Diarrhoea: NR PROM: NR	NR	NR
Abreu et al. [44]	1	NR	NR	Pad free: 25/25 (100) Leak free: NR PROM: NR	Ability to have penetrative sex: 2/7 (29) PROM: IIEF-5	Rectourethral fistula: 0/25 Perineal pain: NR Rectal bleeding: NR Diarrhoea: NR PROM: NR	NR	NR

NR = not reported; PROM = patient-reported outcome measure; IPSS = International Prostate Symptom Score; RTOG = Radiation Therapy Oncology Group; UTI = urinary tract infection; UCLA-EPIC = University of California, Los Angeles-Expanded Prostate Index Composite.

* Ability to have penetrative intercourse.

** Pad-free, leak-free continence; erections sufficient for penetration; absence of clinically significant disease after focal therapy.

### 3.6 Current outcomes in respect of focal therapy in the primary setting

#### 3.6.1 Side effects, complications, and quality of life

Table 6 summarises the morbidity and functional outcome of the studies selected. Median length of hospital stay was 1 d; other perioperative outcomes were poorly reported, with only one study using a standardised classification of these outcomes (Dindo-Clavien classification) [39]. The incidences of the most frequent complications, namely, urinary retention, urinary stricture, and urinary tract infection, ranged from 0% to 17%, from 0% to 5%, and from 0% to 17%, respectively. Only five studies actually reported all of these [25], [29], [30], [35], [39].

Urinary functional outcomes were reported using validated questionnaires in nine studies; physician-reported rates were used in five studies. Using validated questionnaires, the pad-free continence rate varied between 95% and 100%, and the range of leak-free rates was 83–100%.

Erectile function was reported using validated questionnaires in 10 studies and using physician-reported rates in three studies. Considering only trials evaluating focal therapy with *intention to treat*, when validated questionnaires were used, erectile function sufficient for penetration was reported in 54–100% of patients (with or without PDE5-I medication). Physician-reported rates ranged from 58.1% to 85%.

Rectal toxicity was often poorly reported. When it was reported, rates of fistula ranged from 0% to 1%; one series reported one of 41 men suffering grade 3 rectal toxicity conservatively managed as a possible rectourethral fistula [35]. Finally, patient-reported outcomes evaluating overall QOL were uncommonly used in these studies, with only three publications reporting them.

#### 3.6.2 Cancer control

Apart from early feasibility trials (*n* = 6) that verified the effect of tissue ablation by analysis of radical whole-mount prostatectomy specimens, nine series incorporated routine, mandatory, postfocal therapy biopsies in their protocol. In the six early series, residual disease was found in 73 of 74 men who had undergone radical prostatectomy. Although this rate seems excessively high, it should be noted that being early stage 0/1 trials, the main objective was to assess the safety of the sources of energies without actually attempting to ablate all the disease present.

Of the remaining nine series, biopsies were performed only on the treated side in three series; in the other six, biopsy specimens also were taken on the contralateral side. When post-therapy biopsy procedures were routinely offered, clinically significant cancer was present in 0–17% (*n* = 202). When clinically insignificant cancer also was taken into account (excluding one feasibility trial that evaluated safety rather than ablation), 4–50% of men had positive biopsy results after treatment (*n* = 255). When biopsy procedures were offered only *for cause*, overall positive biopsy rates of 13–71% were demonstrated for all types of cancer; when considering all patients enrolled in these series, this percentage range was 3.7–23%. None of these series reported the percentage of significant cancer among patients undergoing a biopsy.

Two series evaluated the presence of residual tumour in the treated area; this amounted to 3–14% when considering only patients undergoing a biopsy and from 1.7% to 3.9% when the denominator was all treated patients.

Biochemical control was reported using Phoenix criteria in five series. Other definitions used were ASTRO (five series), Stuttgart (one series), and Phoenix plus PSA velocity >0.75 ng/ml per year (one series). The results range from 86.2% at 8-yr follow-up (*n* = 318 men) to 60% at 5 yr (*n* = 56) [24], [37].

Only 12 series reported the need for secondary focal treatments, with a range of 0–34%. Salvage local treatments were reported in 14 series with rates of 0–33%. One feasibility trial had higher secondary focal (67%) and salvage treatment (83%); these upper percentages were not considered in the overall range, since the intent to treat was not to destroy all tumour [19].

The progression to metastatic disease is not reported in most of the studies, as the follow-up is too short to have a significant percentage of patients develop metastasis. Nevertheless, when it is indicated, it is extremely low (0–0.3%).

Cancer-specific survival was extremely high in these studies, as expected with the small numbers and short follow-up inherent in almost all reported series; only three studies had a follow-up >5 yr. No man died of PCa after focal therapy in the defined follow-up period. Four men died of other causes in the follow-up period. The very low mortality rate was as expected with the short follow-up and the inclusion of many men with low-risk disease, which has a prolonged natural history.

### 3.7 Current outcomes of focal salvage therapy for failure after radiation therapy

#### 3.7.1 Side effects, complications, and quality of life

The toxicity and QOL outcomes for focal therapy after radiation failure are reported in Table 7 from five published series with a total of 115 men treated. The small numbers considerably limit the generalizability of these findings. Continence, estimated by pad-free rate, was achieved in 87.2–100% of patients. Erectile function was poorly reported, possibly as a result of poor baseline function. However, in three studies (*n* = 82), potency was preserved in 29–40% of previously potent patients [42], [43], [44]. The rate of rectourethral fistula (0–12%) was significantly higher than in the primary cases.

#### 3.7.2 Oncologic outcome

Follow-up was a median range of 17–47 mo. Apart from one feasibility trial, in which the positive biopsy rate for all cancer was 50% in all areas and 33% in the treated area, residual cancer was found in 8–10% of patients using TRUS biopsy [40], [42], [44]. However, this percentage was as high as 44% using TTMB, if considering only patients who had a *for cause* biopsy as the denominator. When considering all patients treated, the positive biopsy rate was 10% [43]. Only one series reported the presence of residual significant cancer, and it showed a rate of 8% [44].

Biochemical disease-free rates in the longest series using the Phoenix criteria were 70% and 54% at 4 and 5 yr, respectively [41], [44]. In one series, the bDFS at 2 yr was significantly lower at 42% using the Stuttgart criteria [43]. Salvage treatment was given to 8–41% of patients, and metastatic disease was diagnosed in 5–20%. Overall survival was 100% in the two series that reported this outcome [40], [44].

## 4 Discussion

This systematic review highlights that when focal therapy is delivered with intention to treat, the perioperative, functional, and disease control outcomes are encouraging.

Although our systematic review was, by its nature, thorough, there were areas that we could not evaluate but that are pertinent to the debate surrounding focal therapy. First, in the light of new findings regarding PCa pathology and natural history, it appears clear that focal therapy should targeted to patients who are likely to benefit from active treatment, whereas men with clinically insignificant disease should be monitored carefully by active surveillance. Specifically, patients with clinically significant disease localised only in one area of the prostate should be considered the optimal candidates for a focal approach.

Second, accurate localisation of disease is essential with mpMRI or novel ultrasound modalities with targeted biopsy of suspicious areas, when available; equally, TTMB may also form part of a rigorous preoperative assessment.

Third, patients treated with an organ-preserving approach must be monitored with strategies similar to active surveillance protocols. Indeed, the presence of significant undetected disease, residual disease, cancer progression, or de novo cancer are all possibilities that mandate active monitoring. However, although the follow-up of men after organ-sparing approaches requires measuring the PSA level, this will not sufficient by itself until validated biochemical measures are developed. Biopsy of the treated and untreated areas are required in the interim, although MRI may play a role in the future if it can be validated for detecting local failure against histologic outcomes.

Fourth, before focal therapy becomes an alternative standard option across the board, it should be highlighted that many issues remain to be addressed, including determining which ablative technology has better functional and oncologic outcome, the margin of normal tissue required, and the long-term disease-control outcomes. In addition, the encouraging results of focal therapy that we report here are the outcomes of a few experienced centres; their generalizability has yet to be proven and training and quality control will be key factors driving the dissemination further.

Finally, we did not address the level of evidence that should drive change. In the studies included, no prospective development study was powered on oncologic outcome, and only two series had a follow-up >5 yr; therefore, no significant conclusion on disease control could be derived. Certainly, high-quality effectiveness studies comparing focal therapy to standard treatments (level 1 evidence) are needed to change practice.

The design of future effectiveness trials comparing focal to whole-gland therapies is debatable. Some have argued for long-term mortality outcomes; however, if this were the case, progress and change would be prohibitively delayed because the studies required for mortality outcomes are likely to take 15–20 yr to deliver data and unlikely to recruit. If mortality is out of reach and biochemical outcomes are implausible, at least for the time being, then other more pragmatic outcomes will be necessary. These are likely to include (1) treatment-specific and overall QOL measures, (2) local cancer control measured as absence of significant PCa, (3) rate of additional systemic therapy, and (4) cost effectiveness.

The first pragmatic outcome relates to treatment-related side effects and can be relatively well captured in the short term using validated questionnaires. These are principally directed at genitourinary and bowel-associated outcomes and have been used in the evaluation of all the interventions under consideration. The second pragmatic outcome relates to effectiveness of local cancer control. Histologic confirmation of complete ablation within the treated area appears to be essential when a man is treated with focal therapy, given the uncertainty of PSA follow-up. However, TRUS biopsy would have the same inherent random and systematic sampling errors when used after treatment and may not be reliable in determining the absence of residual disease. At present, TTMB appears to be one of a number of accurate tools for confirming the effectiveness of the treatment, as the possibility of missing significant PCa is <5% with this technique [72]. In contrast, some investigators have used mpMRI to assess recurrence and the initial results seem promising. In two studies of patients treated with focal HIFU, one including 20 patients and the other 41, no significant cancer was found in the treated area when mpMRI did not show signs of residual disease [30], [35].

The third pragmatic outcome, use of additional systemic therapy, could be regarded as the only acceptable outcome measure that would cover focal therapy and standard care objectively. This outcome should clearly be separated from the need for additional local treatment or local failure. Indeed, secondary ablation with the same or a different energy applied in a focal manner probably should not be incorporated in this pragmatic outcome as a failure although the application of whole-gland therapy using any modality would constitute failure at that point in time. In other words, this pragmatic outcome should count as failure when there is a change in strategy from focal to whole-gland ablation or the direct shift to systemic therapy; this would allow realistic comparison with standard treatments. This would have to be verified by evidence that men who have second or even third focal treatments do not suffer worse genitourinary and disease-control rates compared to men who have radical treatments. Finally, the effectiveness of each therapy should be balanced with its cost to allow objective comparisons between different active treatments.

## 5 Conclusions

Although focal therapy may be regarded as an alternative to active surveillance by many physicians, it should not be proposed to patients with confirmed, clinically insignificant, low-risk disease who are unlikely to benefit from any form of treatment and in whom even focal therapy would be regarded as an overtreatment. Robust effectiveness studies are now required to compare focal therapy to radical therapy in men with clinically significant PCa.

  ***Author contributions*****:** Massimo Valerio and Hashim U. Ahmed had full access to all the data in the study and take responsibility for the integrity of the data and the accuracy of the data analysis.  

*Study concept and design*: Valerio, Ahmed, Emberton, Lawrentschuk, Lazzeri, Montironi, Nguyen, Trachtenberg, Polascik.

*Acquisition of data*: Valerio, Ahmed.

*Analysis and interpretation of data*: Valerio, Ahmed, Emberton, Lawrentschuk, Lazzeri, Montironi, Nguyen, Trachtenberg, Polascik.

*Drafting of the manuscript*: Valerio, Ahmed.

*Critical revision of the manuscript for important intellectual content*: Valerio, Ahmed, Emberton, Lawrentschuk, Lazzeri, Montironi, Nguyen, Trachtenberg, Polascik.

*Statistical analysis*: None.

*Obtaining funding*: None.

*Administrative, technical, or material support*: Emberton, Lawrentschuk, Lazzeri, Montironi, Nguyen, Trachtenberg, Polascik.

*Supervision*: None.

*Other* (specify): None.  

***Financial disclosures*****:** Massimo Valerio certifies that all conflicts of interest, including specific financial interests and relationships and affiliations relevant to the subject matter or materials discussed in the manuscript (eg, employment/affiliation, grants or funding, consultancies, honoraria, stock ownership or options, expert testimony, royalties, or patents filed, received, or pending), are the following: The SICPA foundation supports the ongoing fellowship and PhD program of M. Valerio. M. Emberton and H.U. Ahmed would like to acknowledge funding from the Medical Research Council (UK), the Pelican Cancer Foundation charity, Prostate Cancer UK, St Peters Trust charity, Prostate Cancer Research Centre, the Wellcome Trust, National Institute of Health Research-Health Technology Assessment program, and the US National Institutes of Health National Cancer Institute. M. Emberton receives funding in part from the UK National Institute of Health Research UCLH/UCL Comprehensive Biomedical Research Centre. M. Emberton and H.U. Ahmed receive funding from USHIFU, GSK and Advanced Medical Diagnostics for clinical trials. M. Emberton is a paid consultant to Steba Biotech and USHIFU. Both have previously received consultancy payments from Oncura/GE Healthcare and Steba Biotech.  

***Funding/Support and role of the sponsor***: None.  

***Acknowledgment statement*****:** The authors acknowledge the participation of the following investigators: Rajiv Chopra, Cosimo De Nunzio, Louise Dickinson, Shigeo Horie, Uri Lindner, Stephan Madersbacher, Caroline M. Moore, Rémi Souchon, Osamu Ukimura, John F. Ward, and Alexandre R. Zlotta.
